# Skin microbiome analysis of a junctional epidermolysis bullosa patient treated with genetically modified stem cells

**DOI:** 10.1111/ddg.15776

**Published:** 2025-06-12

**Authors:** Alexander Dermietzel, Burcu Tosun, Mathilde Nguyen, Kai Wessel, Luise Rauer, Avidan U. Neumann, Tobias Hirsch, CK‐CARE study group, Claudia Traidl‐Hoffmann, Matthias Reiger, Claudia Hülpüsch, Maximilian Kueckelhaus

**Affiliations:** ^1^ Department of Plastic Surgery University Hospital Muenster Muenster Germany; ^2^ Department of Plastic Reconstructive and Aesthetic Surgery Hand Surgery Fachklinik Hornheide Muenster Germany; ^3^ Department of Plastic and Reconstructive Surgery Institute for Musculoskeletal Medicine University Muenster Muenster Germany; ^4^ Department for Plastic Reconstructive and Aesthetic Surgery Hand Surgery Rotenburg Rotenburg Germany; ^5^ Institute of environmental medicine and integrative health Faculty of Medicine University of Augsburg Augsburg Germany; ^6^ Institute of Environmental Medicine Helmholtz Munich Augsburg Germany; ^7^ CK CARE Christine Kühne Center for Allergy Research and Education Davos Switzerland

**Keywords:** Junctional epidermolysis bullosa, skin microbiome, Staphylococcus aureus

## Abstract

**Background and Objective:**

Junctional epidermolysis bullosa (JEB) is a subtype of epidermolysis bullosa caused by mutations in the *LAMB3* gene. We treated a patient with JEB using genetically corrected autologous epidermal cultures retrovirally transduced with the functional *LAMB3* gene sequence. The objective of this study was to analyze the skin microbiome of this patient, with a particular focus on transgenic skin, and to compare the findings to the skin microbiome of healthy controls and patients with atopic dermatitis and well‐documented microbial dysbiosis.

**Patients and Methods:**

Skin microbiome analysis was performed on a JEB patient 72 months after combined gene and stem cell therapy. Skin swabs from age‐matched healthy controls and atopic dermatitis patients were included from the ProRaD study of CK‐CARE.

**Results:**

The transgenic skin had comparably high relative and absolute *Staphylococcus (S.) aureus* abundance to blistering and non‐blistering skin of the JEB patient, while the total bacterial load was lower. In blistering skin of the JEB patient, higher bacterial load was driven by *S. aureus*.

**Conclusions:**

Our investigation confirms a unique microbiome composition in JEB, characterized by *S. aureus* driven bacterial overgrowth. The dysbiosis was not reversed in transgenic, non‐blistering skin areas. However, the transgenic skin demonstrates stability in an environment of bacterial dysbiosis.

## INTRODUCTION

Junctional epidermolysis bullosa (JEB) is a severe genetic subtype of the epidermolysis bullosa (EB) group, caused by mutations in the *LAMB3* gene encoding the β3 chain of laminin 332.^1^, ^2^, ^3^, ^4^ Affected patients suffer from blistering of the skin caused by minor mechanical stress. Severe blistering leads to scarring, infection and progressively debilitating advance of the disease up to an early death. Over 40% of patients die before reaching adulthood.^5^, ^6^, ^7^

Until 2015, no definitive treatment existed for inherited JEB. Our research team treated a young patient suffering from JEB, who had about 80% of his epidermis destroyed. After all established therapies had failed, our team decided to take an experimental approach and transplant skin made from genetically modified stem cells onto the wound surfaces. Stem cells were harvested via a skin biopsy and transfected by using a retroviral vector containing the functional gene sequence for the *LAMB3* gene.^8^, ^9^, ^10^

Before transplantation, superinfection with *Staphylococcus (S.) aureus* persisted despite regular antibiotic treatment. The infection progressively worsened, culminating in severe sepsis with early‐stage organ failure at the time of transplantation. Transplantation of the genetically transfected skin was a success. Our patient recovered and was discharged from the hospital. Since then, a 5‐year follow‐up has demonstrated long‐term stability of the entire transgenic epidermis, with no recurrence of blistering in the transplanted areas. These findings have been reported in previous publications by our group.^8^, ^11^, ^12^

Human skin is not only the body's largest organ and a barrier against environmental influences, but is also protected by its own cutaneous microbiome. The microbiome aids as an additional barrier to the physical and chemical barrier towards environmental influences and protects the skin against diseases^13^, ^14^. It is a complex and dynamic community of bacteria, fungi, and other microorganisms that live on the surface of the skin. It plays an important role in maintaining skin health, protecting against pathogens, regulating the skin's immune system and is unique to each individual. It can be influenced by factors such as age, sex, lifestyle, environment, diseases, and medical treatments.^15^

Dysbiosis of the skin microbiome is associated with skin diseases such as atopic dermatitis (AD), in which an increased abundance of *S. aureus* can exacerbate disease severity, for example through toxin production.^16^, ^17^, ^18^, ^19^, ^20^

The exact microbiome of EB has not been extensively studied. However, research has shown that individuals with EB have an altered microbiome compared to healthy individuals, with an overabundance of *S. aureus* and an overall decrease in the diversity of skin microbes.^21^, ^22^

Due to the preceding results our research team decided to further investigate changes intra‐individually in the skin microbiome of our patient treated with genetically corrected autologous epidermal cultures and compare it to the skin microbiome of healthy controls and AD patients.

## OBJECTIVE

The objective of this research was to analyze the skin microbiome of a JEB patient treated with genetically corrected autologous epidermal cultures. Of particular interest was the microbial composition of the transgenic skin area compared to the surrounding blistering and non‐blistering skin area of the JEB patient 72 months after combined gene and stem cell therapy.

In addition, the skin microbiome of this JEB patient was compared with the microbial composition of the skin of healthy controls and AD patients.

## PATIENTS AND METHODS

All study methods followed the *Declaration of Helsinki*. Ethical approval for this study was granted by the ethics committee of the *University of Muenster* (Reference: 2020‐804‐f‐S). Data of AD patients was included in the study from the *Prospective longitudinal study to investigate the remission phase in patients with atopic dermatitis* (ProRaD study) which were approved by the respective local ethics committee of Zurich, Switzerland (BASEC 2016‐00301, ClinicalTrials.gov Identifier: NCT04240522) and the local ethics committee of the Technical University of Munich (112/16S) and Bonn (ProRAD 232/15). The ProRaD study investigates biomarkers and the cutaneous microbiome of AD patients in remission in a longitudinal prospective setting.^23^

### Study population

**FIGURE 1:**
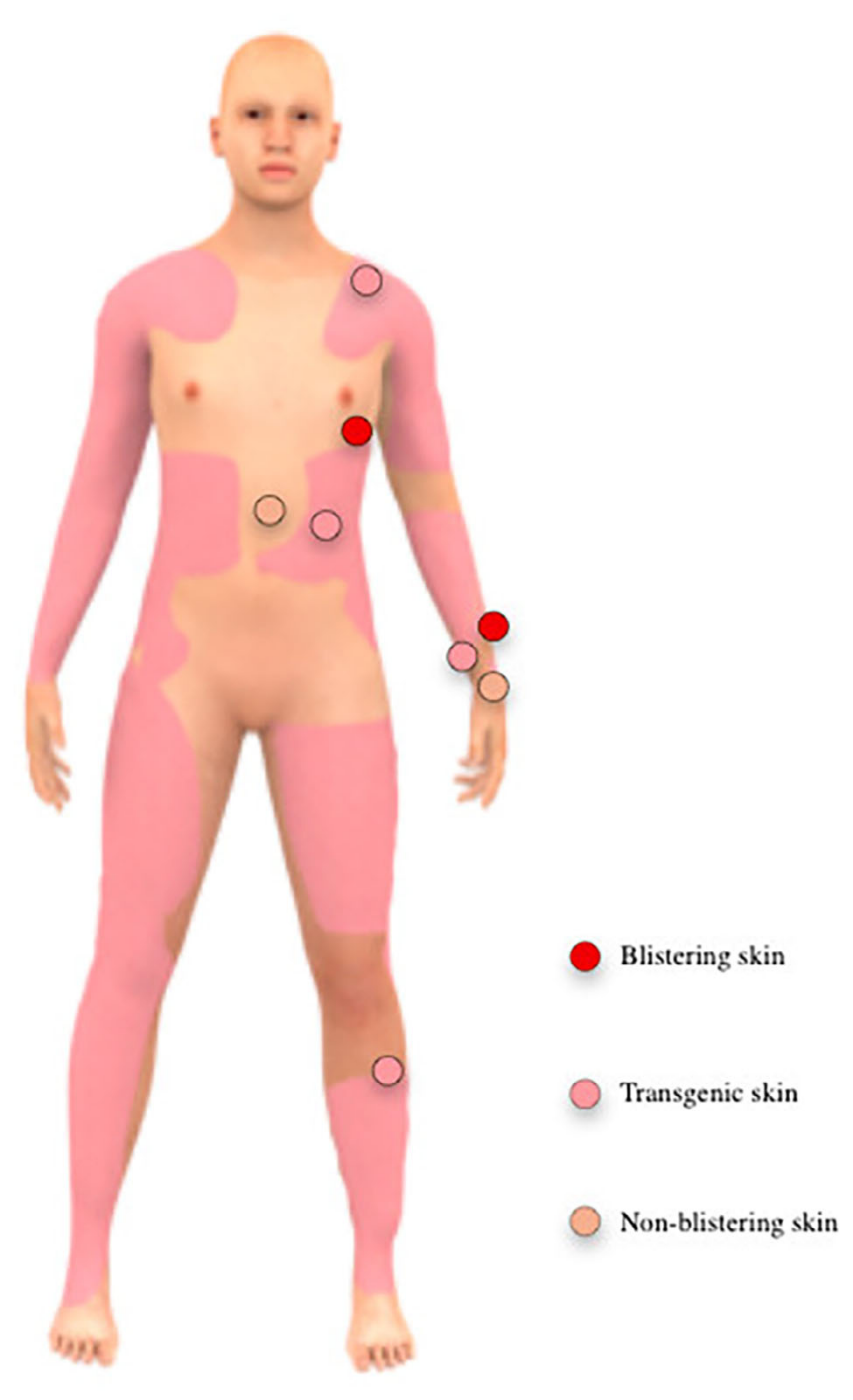
Skin qualities of JEB patient. Blistering skin, non‐blistering skin and transgenic skin areas of JEB patient. Lower arm, wrist, lower leg, thorax, abdomen and shoulder were sampled for microbiome analysis.

Skin microbiome analysis was performed on a JEB patient 72 months after combined gene and stem cell therapy. We sampled the JEB patient by swabbing blistering skin, non‐blistering skin and transgenic skin for microbiome analysis. Skin swabs were collected from lower arm, wrist, lower leg, thorax, abdomen and shoulder, as shown in Figure 1. Per skin site, replicates were taken. Furthermore, three swabs from one healthy individual were taken in parallel to the JEB patient to be able to combine the data with another dataset containing controls to put the skin microbiome findings of the JEB patient into perspective.

As controls, four healthy children and ten healthy adults were included from the ProRaD study population. To compare the microbial composition of JEB with a disease characterized by a known microbial dysbiosis, additional samples from the skin of the antecubital fossa as a typical affected body site of AD patients were included from the ProRaD study, as described in detail in Table 1 (5 non‐lesional sample from children, 5 lesional samples from children, 10 non‐lesional samples from adults and 10 lesional samples from adults).

**TABLE 1:** Sample overview. The number of samples per health and skin status are summarized in the table.

Health status	Skin status	Age group	Location	Study origin	Available samples per analysis Total (qPCR, NGS)
JEB	Ebb	Child	Lower arm, breast	Münster	6 (6, 3)
JEB	Ebnb	Child	Hand, wrist, belly	Münster	6 (6, 5)
JEB	T	Child	Lower arm, lower leg, belly, shoulder	Münster	8 (8, 8)
HE	NL	Adult	Breast, arm, shoulder	Münster	3 (3, 3)
HE	NL	Child	Antecubital fossa	ProRaD	4 (4, 4)
HE	NL	Adult	Antecubital fossa	ProRaD	10 (10, 10)
AD	NL	Child	Antecubital fossa	ProRaD	5 (5, 5)
AD	LS	Child	Antecubital fossa	ProRaD	5 (5, 5)
AD	NL	Adult	Antecubital fossa	ProRaD	10 (10, 10)
AD	LS	Adult	Antecubital fossa	ProRaD	10 (10, 10)

*Abbr*.: AD, atopic dermatitis; Ebb, junctional epidermolysis bullosa blistering skin; Ebnb, junctional epidermolysis bullosa non‐blistering skin; HE, healthy controls; JEB, junctional epidermolysis bullosa; LS, lesional skin; NL, non‐lesional skin; NGS, next‐generation sequencing; T, junctional epidermolysis bullosa transgenic skin

### Data analysis

*Sampling*. For 16S rRNA gene amplicon sequencing, samples of all datasets were prepared, as previously published.^24^ In brief, skin swabs (Sigma‐swab, MWE, Corsham, England) were taken and stored in 500 µL of Stool DNA Stabilizer solution (Stratec, Berlin, Germany).

*DNA extraction*. The DNA was extracted with the QIAamp UCP Pathogen kit (Qiagen: Hilden, Germany) as previously published.^24^

### Next generation sequencing preparation

*Amplification*. The V1–V3 region of the 16S rRNA gene was amplified using the 27F‐YM (5‐AGAGTTTGATYMTGGCTCAG‐3) and 534R (5‐ATTACCGCGGCTGCTGG‐3) primers. Barcodes were added in a second PCR step.

*Library preparation*. Thereafter, AMPure XP beads (Beckman Coulter, Fullerton, CA, USA) were used for amplicon purification. Samples were sequenced with the Illumina MiSeq^®^ platform (Illumina Inc., San Diego, CA, USA) using 2 × 300 bp paired‐end reads (MiSeq^®^ Reagent Kit v3 600 cycles; Illumina Inc.).

*Bioinformatics*. Denoising of the sequences was performed with DADA2^25^ and annotation with AnnotIEM^26^. MicrobIEM^27^ was used for removing contaminants, singletons, and samples with low reads. The number of samples as stated in Table 1 passed quality control.

### Merging of datasets

Despite using the same protocols for all samples, the samples from the Münster and ProRaD study were sequenced in two different batches. The JEB samples and the ProRaD samples including the additional samples from AD patients and healthy controls were sequenced on different sequencing runs. To reduce biases introduced by the sequencing run and the impact of sparse sequences, the relative abundance of each species was calculated per sample. The top ten species per sequencing run and dataset were extracted and merged, resulting in final analysis dataset containing thirteen species. All others were summarized as “others”.

### Quality control of dataset merging

After analysis of the JEB microbiome a further comparison to AD and healthy age matched controls was performed. To confirm whether the method chosen for the merging of datasets from two sequencing runs was suitable, the global microbiome between the healthy adult individuals of both datasets was compared, as this was the only patient group which was present in both sequencing runs. As the samples clustered together in the global microbiome, the method was thought to be suitable for comparing the samples from the JEB patient with the samples from AD patients and age‐matched healthy controls (online supplementary Figure S1).

### Quantification via qPCR

To quantify the absolute bacterial load, the 16S rRNA gene was used as a proxy. For *S. aureus* quantification, the unique *S. aureus* gene *nuc* was used. The qPCR was carried out via a TaqMan assay using the following primers and probes:

*S. aureus*:

‐ *Forward primer*: GTTGCTTAGTGTTAACTTTAGTTGTA
‐ *Reverse primer*: AATGTCGCAGGTTCTTTATGTAATTT
‐ *Probe*: FAM‐AAGTCTAAGTAGCTCAGCAAATGCA‐BHQ1^28^

*16S rRNA gene copies*:

‐ *Forward primer*: TGGAGCATGTGGTTTAATTCGA
‐ *Reverse primer*: TGCGGGACTTAACCCAACA
‐ *Probe*: Cy5‐CACGAGCTGACGACARCCATGCA‐BHQ2 (Eurogentec S.A., Seraing, Belgium)^29^

The reactions were performed in 10 µL final volume using the PerfeCTa Multiplex qPCR ToughMix (Quantabio, Beverly, MA, USA) with a 100 nM concentration for each primer and probe in the multiplex setup. Following a 2‐minute denaturation–activation step at 95°C, 45 cycles were conducted, each consisting of a 15‐second denaturation at 95°C and a 60‐second annealing–elongation at 60 °C, using a CFX384 Real‐Time System (Bio‐Rad Laboratories, Inc., Hercules, CA, USA). The quantity cycles (Cqs) were determined as the average of independent triplicates.

## RESULTS

### Skin microbiome of transgenic skin of JEB patient

**FIGURE 2:**
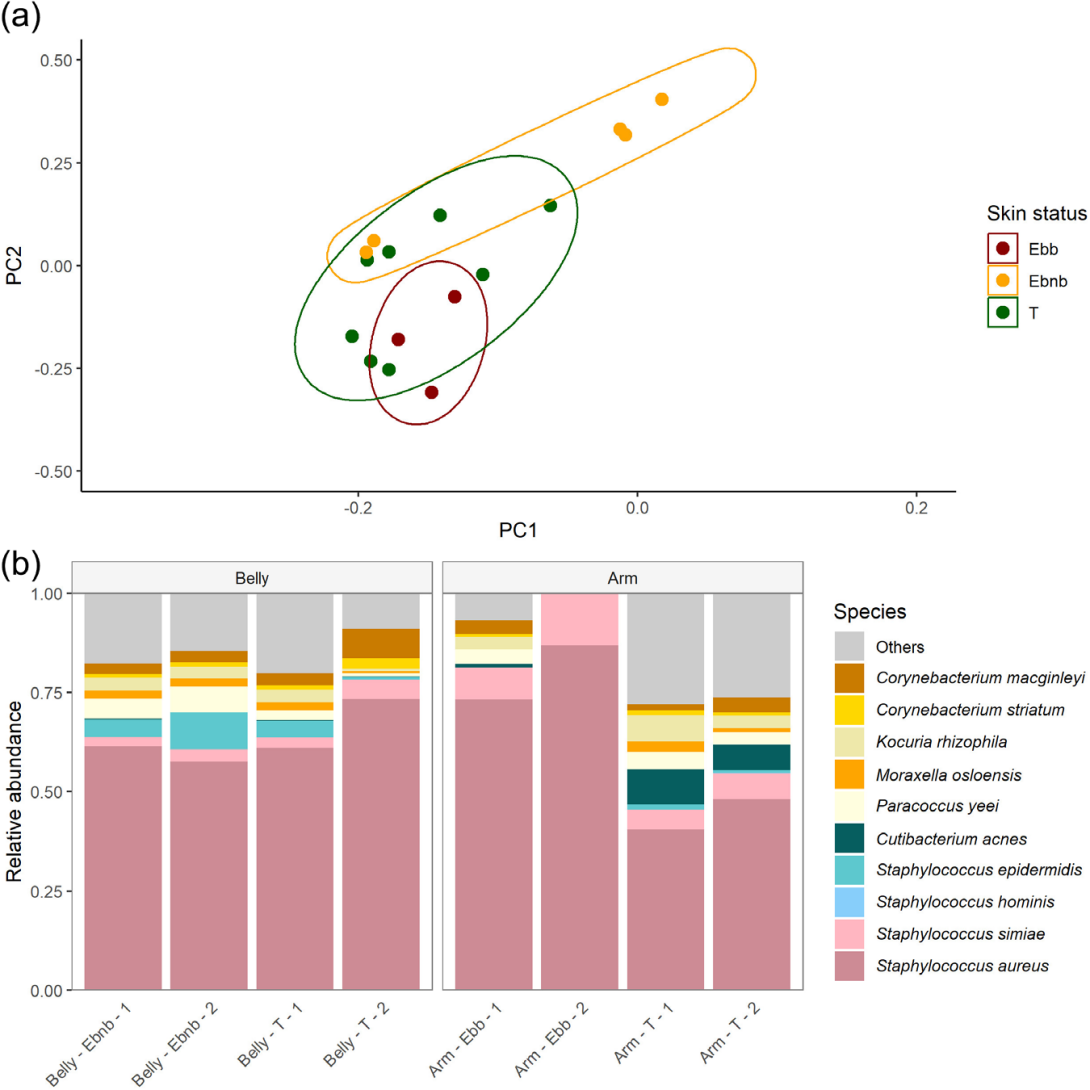
The skin microbiome of the transplant resembles the surrounding non‐blistering skin of the JEB patient. Global microbiome of transgenic skin clustered between blistering skin and non‐blistering skin. (a) Global skin microbiome represented with a principal coordinate analysis (PCoA) based on the Bray‐Curtis distance. (b) Skin microbiome taxonomy of blistering, non‐blistering and transgenic skin at arm and belly. Transgenic skin has a similar skin microbiome as non‐blistering skin at the belly while on the arm the S. aureus relative abundance was reduced. *Abbr*.: Ebb, junctional epidermolysis bullosa blistering skin; Ebnb, junctional epidermolysis bullosa non‐blistering skin; T, junctional epidermolysis bullosa transgenic skin

The global skin microbiome of the JEB patient differed depending on skin status –blistering, non‐blistering, and transgenic areas. Blistering skin (Ebb) and non‐blistering skin (Ebnb) were the most distinct in terms of the global skin microbiome, while the transgenic (T) skin microbiome was positioned in between non‐blistering and blistering skin, as shown in Figure 2a.

The ten most abundant species were present in all samples independent from skin status and body location. Per location, mostly two replicates were available which had a high similarity (online supplementary Figure S2). Generally, *S. aureus* was the most abundant species. However, the individual sample composition varied (online supplementary Figure S2).

To exclude the influence of the body location on the skin microbiome composition, the microbiome composition of the skin status was compared only within one body location where different skin status were available (arm, belly). As shown in Figure 2b, the arm had a higher relative abundance of *S. aureus* in the blistering skin compared to the transgenic neighboring skin, whereas no difference was found between the skin microbiome in non‐blistering skin and transgenic skin in the belly, which were both dominated by *S. aureus*.

### *Staphylococcus aureus* driven increase in bacterial load in blistering skin

**FIGURE 3:**
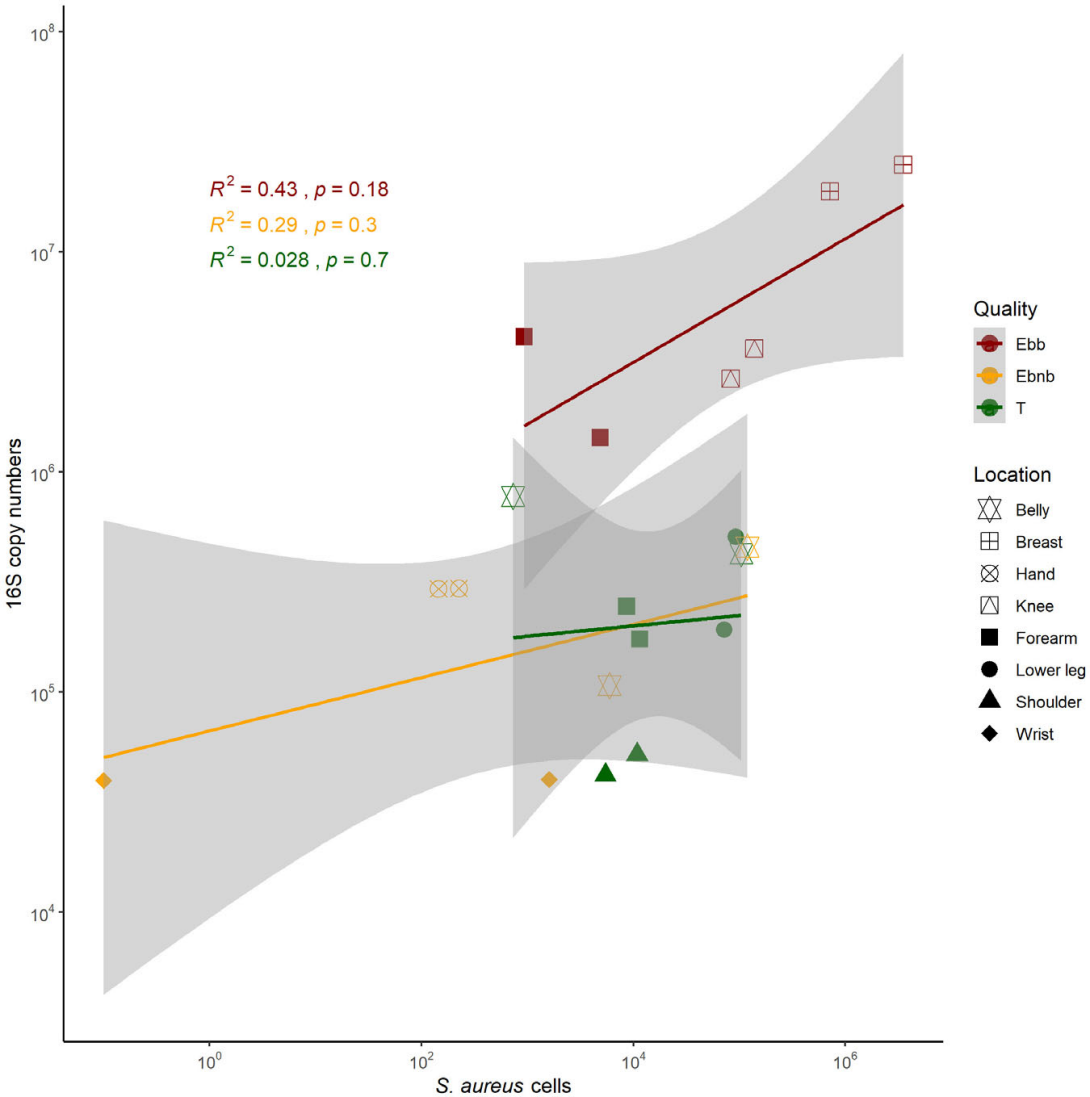
*Staphylococcus aureus* driven bacterial overgrowth in blistering skin of JEB patient. qPCR of the 16S rRNA as proxy for bacterial cell numbers with highest load in blistering skin. High bacterial load driven by *S. aureus* cell number in blistering skin shown by a strong but non‐significant correlation. Spearman correlation was performed for each type of samples. Sampling location is indicated by shape. *Abbr*.: HE, healthy controls; AD, atopic dermatitis; JEB, junctional epidermolysis bullosa; NL, non‐lesional skin; LS, lesional skin; Ebb, junctional epidermolysis bullosa blistering skin; Ebnb, junctional epidermolysis bullosa non‐blistering skin; T, junctional epidermolysis bullosa transgenic skin; HE child, 4 NGS, 4 qPCR; HE adult, 10 NGS, 10 qPCR; HE adult*, 3 NGS, 3 qPCR; AD NL child, 5 NGS, 5 qPCR; AD NL adult, 10 NGS, 10 qPCR; AD LS child, 5 NGS, 5 qPCR; AD LS adult, 10 NGS, 10 qPCR; Ebnb, 5 NGS, 6 qPCR; Ebb, 3 NGS, 6 qPCR; T, 8 NGS, 8 qPCR. *Healthy adult from EB dataset.

In the blistering skin areas of the JEB patient, both *S. aureus* absolute cell numbers determined by qPCR and 16S copy number as a proxy for bacterial cell numbers were higher than in non‐blistering and transgenic skin (online supplementary Figure S2, S3 and S4). The higher bacterial load was driven by *S. aureus* cells, as shown in Figure 3. Even though *S. aureus* absolute abundance was still high in transgenic skin, the absolute bacterial numbers were lower than in blistering skin, at the same level of non‐blistering skin.

### Specific skin microbiome of JEB

**FIGURE 4:**
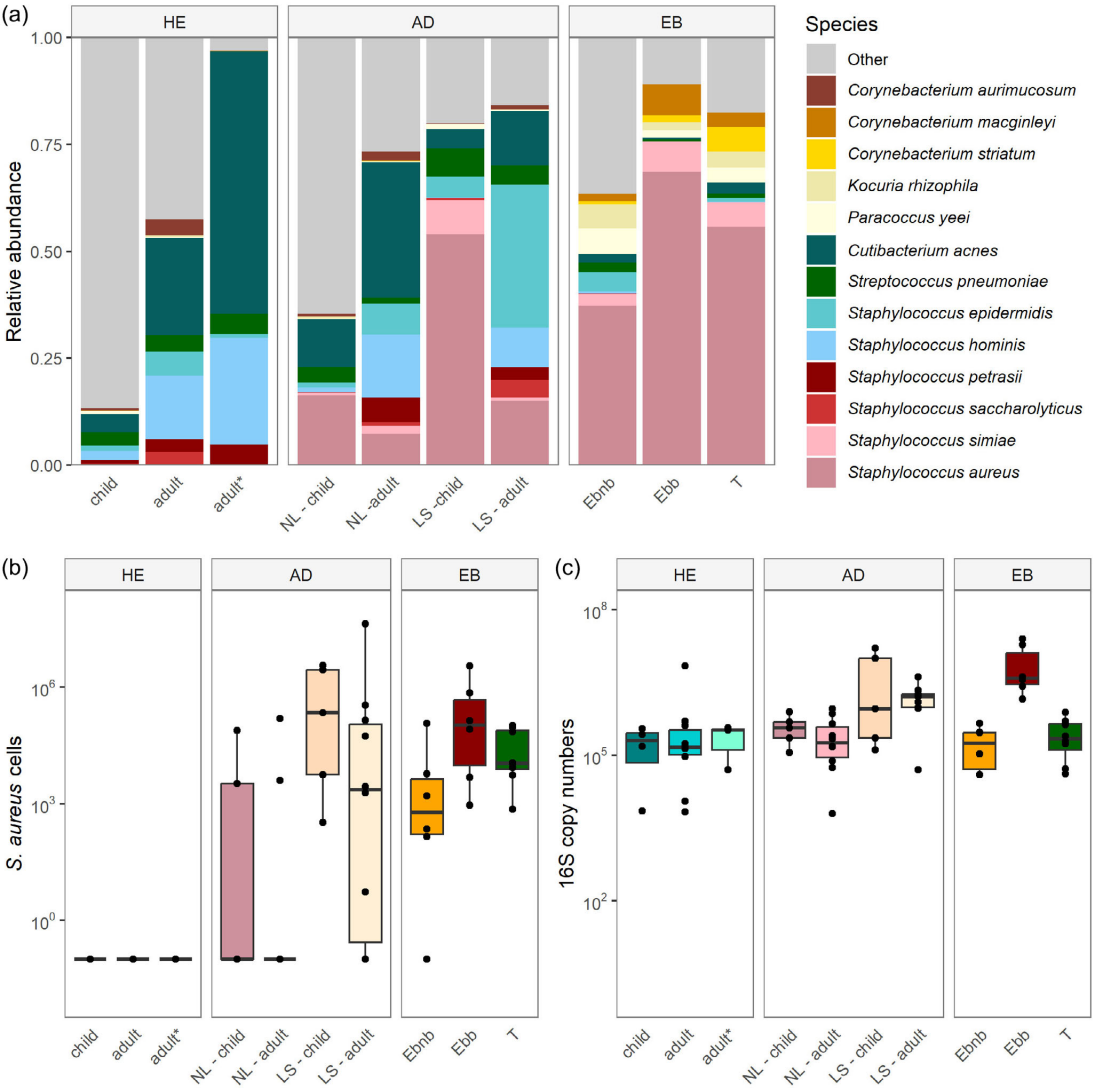
The skin microbiome of the JEB patient is distinct compared to healthy controls and AD patients. (a) Top 13 species of HE, AD and EB differ. Furthermore, the skin status shows a difference in the microbial composition. (b) The absolute cell numbers of S. aureus measured via qPCR especially of blistering skin are similar to S. aureus load in AD lesional skin. The absolute number of bacterial cells measured via qPCR especially in blistering skin were at similar levels as bacterial cells detected in AD lesional skin samples.

Comparing the top ten species of the JEB patient with healthy controls and AD patients revealed strong differences between the health groups. While the skin of the JEB patient was highly dominated by *S. aureus*, the skin of healthy children and adults harbored a variety of *Staphylococcus, Streptococcus* and *Cutibacterium* species. Interestingly, the skin of JEB patient specifically harbored *Corynebacterium (C.) macginleyi, C. striatum* and *Kocuria rhizophila* while other typical commensals like *S. epidermidis, S. hominis*, and *Cutibacterium acnes* were reduced. The relative abundance of *S. aureus* on the skin of the JEB patient was even higher than in lesional samples of AD patients (Figure 4). Additionally, the absolute levels of *S. aureus* and bacterial cells were similar to those detected in lesional skin of AD patients (Figure 4b,c). Even though high numbers of *S. aureus* cells were detected in transgenic skin, absolute bacterial load was similar to non‐blistering skin (Figure 4b,c).

## DISCUSSION AND LIMITATIONS

In this study we could show that non‐blistering, blistering and transgenic skin of a JEB patient had a dysbiosis towards *S. aureus* in relative and absolute numbers. Blistering skin revealed an *S. aureus* driven bacterial overgrowth. The skin microbiome of the JEB patient was distinct from healthy controls and AD patients.

When comparing healthy skin with chronic wounds, various studies in the past have shown a reduced microbial diversity and high incidence of *S. aureus* in chronic wounds in general.^21^, ^30^ Patients with atopic dermatitis have reduced levels of beneficial bacteria, such as *S. epidermidis*, and increased levels of other microbial species, including *S. aureus*, *Pseudomonas aeruginosa*, and Malassezia species.^15^, ^31^ Studies have shown that the relative and absolute abundance of *S. aureus* correlates with the severity of the disease.^24^, ^32^

Non‐blistering and wounded skin of patients with recessive dystrophic EB show a significantly reduced diversity and a high proportion of *S. aureus* in blistering and non‐blistering skin. Reimer‐Taschenbrecker et al. observed a dominance of *S. aureus* which, depending on age, first affects the injured/blistering skin and later the non‐injured/non‐blistering skin^1^.

In these cases, severity of the disease and wound burden significantly correlates positively with *S. aureus* colonization similar to other skin diseases such as AD. A reason for general dysbiosis and abundance of *S. aureus* colonization could be the necessary wound dressings of blistering areas, which often overlap and cover non‐blistering areas. This creates a positive environment for dissemination of *S. aureus* across the skin surface. Blistering skin areas are initially affected and wound dressings can promote bacterial spreading. Supporting this hypothesis, Horev et al. (2023) showed a higher abundance of *S. aureus* in EB wounds after the application of wound dressings. Ninety days after wound dressing treatment, children with EB dystrophica and EB simplex showed a significantly higher incidence of *S. aureus* in the blistering skin areas.^33^

Repetitive antibiotic treatment is also necessary in case of wound infection. This too creates a selective dysbiosis of the cutaneous microbiome and a potential formation of multiresistant bacteria such as methicillin‐resistant *S. aureus (MRSA)*.^34^, ^35^

Our JEB patient´s skin microbiome differs depending on skin status. The microbiome of transgenic skin is clustered between blistering and non‐blistering skin. Blistering skin shows a higher proportion of *S. aureus* compared to non‐blistering and transgenic skin, whereas transgenic skin and non‐blistering skin are similar. As the microbiome differs across the body's surface, sampling of all three tissue types (blistering, non‐blistering, and transgenic) was not trivial in our patient and reduced the number of possible testing areas.

We could show a distinct global microbiome of the investigated JEB patient after transgenic skin transplantation from the global microbiome of healthy controls and AD. Absolute bacterial cell numbers in blistering skin were at similar levels detected in AD patients from the ProRaD study.

Despite this dysbiosis, transgenic areas remained stable and showed no clinical signs of eczema or blistering. These findings confirm the genetic stability of vector‐transfected skin regions, indicating sustained resistance to blister recurrence and microbial dysbiosis.

Limitations of this study are the small sample size. Our patient is the only one to date to have received the aforementioned treatment. Sampling was restricted to areas where all three tissue types – listering, non‐blistering, and transgenic – coexisted, thereby reducing the number of feasible biopsy sites and limiting the overall sample size. Therefore, no statistical test for body regions is possible. To confirm our data, independent replicates were performed and showed reliable results. However, intra‐individual differences, as well as environmental influences, are possible.

Future investigations should focus on follow‐up studies of the microbiome in the transplanted JEB patient. Such studies could clarify whether the microbiome is affected by physiological changes, for example hormonal shifts occurring during puberty or adolescence. Furthermore, environmental testing should be expanded to the family of our patient and examine if family members are colonized by the same *S. aureus* as our patient.

## FUNDING

We thank the *Christine Kühne‐Center for Allergy Research and Education* (CK‐CARE), Davos, Switzerland for funding of the “Prospective longitudinal study to investigate the remission phase in patients with atopic dermatitis”. No additional funding was received for conducting this study.

## CONFLICT OF INTEREST STATEMENT

None.
